# Species specific and environment induced variation of δ^13^C and δ^15^N in alpine plants

**DOI:** 10.3389/fpls.2015.00423

**Published:** 2015-06-05

**Authors:** Yang Yang, Rolf T. W. Siegwolf, Christian Körner

**Affiliations:** ^1^Key Laboratory for Plant Diversity and Biogeography of East Asia, Kunming Institute of Botany, Chinese Academy of SciencesKunming, China; ^2^Institute of Botany, University of BaselBasel, Switzerland; ^3^Lab for Atmospheric Chemistry, Paul Scherrer InstituteVilligen, Switzerland

**Keywords:** biodiversity, carbon, nitrogen, N_2_ fixation, soil, stable isotope

## Abstract

Stable carbon and nitrogen isotope signals in plant tissues integrate plant-environment interactions over long periods. In this study, we hypothesized that humid alpine life conditions are narrowing the scope for significant deviations from common carbon, water and nitrogen relations as captured by stable isotope signals. We explored the variation in δ^13^C and δ^15^N in 32 plant species from tissue type to ecosystem scale across a suite of locations at c. Two thousand five hundred meter elevation in the Swiss Alps. Foliar δ^13^C and δ^15^N varied among species by about 3–4‰ and 7–8‰ respectively. However, there was no overall difference in means of δ^13^C and δ^15^N for species sampled in different plant communities or when bulk plant dry matter harvests of different plant communities were compared. δ^13^C was found to be highly species specific, so that the ranking among species was mostly maintained across 11 habitats. However, δ^15^N varied significantly from place to place in all species (a range of 2.7‰) except in Fabaceae (*Trifolium alpinum*) and Juncaceae (*Luzula lutea*). There was also a substantial variation among individuals of the same species collected next to each other. No difference was found in foliar δ^15^N of non-legumes, which were either collected next to or away from the most common legume, *T. alpinum*. δ^15^N data place Cyperaceae and Juncaceae, just like Fabaceae, in a low discrimination category, well separated from other families. Soil δ^15^N was higher than in plants and increased with soil depth. The results indicate a high functional diversity in alpine plants that is similar to that reported for low elevation plants. We conclude that the surprisingly high variation in δ^13^C and δ^15^N signals in the studied high elevation plants is largely species specific (genetic) and insensitive to obvious environmental cues.

## Introduction

Based on the correlation between the discrimination of the heavy ^13^C isotope during CO_2_ gas exchange and habitat conditions, δ^13^C has become an important ecological index of plant carbon and water relations and photosynthesis performance (Dawson et al., 2002). In C_3_ plants, ^13^C discrimination can be altered by the combined effect of stomatal control and the activity of the carboxylating enzyme, Rubisco, both under either genetic or environmental control (Geber and Dawson, 1990; Vitousek et al., 1990; Guehl et al., 1995). δ^15^N signals in plant tissues have been shown to reflect the dominant way of N acquisition by plants, with the degree of symbiotical N_2_ fixation and mycorrhizal associations (e.g., ectomycorrhizal and arbuscular mycorrhizal species) contributing most to the variation in ^15^N abundance in plants (Evans, 2001; Dawson et al., 2002; Craine et al., 2009). In addition, the δ^15^N signal in plant tissues is also related to the N-forms available in the soil, which reflects the isotopic fractionations during biogeochemical processes (Högberg, 1997).

High elevation alpine habitats are characterized by cold climate, low partial pressure of ambient CO_2,_ and N limitation of growth (Körner, 2003), all likely to affect δ^13^C and δ^15^N in characteristic ways. For instance, a cross-cutting trend associated with increasing elevation in humid regions (tested for large enough ranges of elevation) is a reduction of the discrimination against ^13^C, interpreted as an increased efficiency of carbon capture in high elevation plants (Körner et al., 1988, 1991). More recent broad samplings of congeneric species revealed that the reduced over all ^13^C discrimination with elevation is associated with atmospheric pressure (its various side effects) and is not related to reduced temperature (Zhu et al., 2010; Zhou et al., 2011), matching results of earlier gas exchange works that showed that temperature exerts minor influences on carbon uptake in alpine plants (Körner, 1982; Körner and Diemer, 1987). Seeming deviations from this pattern had been reported when elevational gradients were confounded with moisture gradients or when the elevational gradients explored were too narrow so that local peculiarities of soil conditions or in genotype overtopped the elevation signal (Körner, 2007). On the other hand, a wider range of interspecific foliar δ^15^N was observed in cold ecosystem (e.g., arctic tundra) compared to other ranges in warmer region (Nadelhoffer et al., 1996; Craine et al., 2009). In alpine ecosystems, microclimate and biota are characterized by small-scale variability over very short distances related to topography (Scherrer and Körner, 2010). These topography-related patterns at high elevation can also produce considerable variations in soil moisture and nutrient availability, impacting plant performance and community composition (Körner, 2003). The influence of topography on the distribution of plant species (May and Webber, 1982), on the above-ground and below-ground productivity (Billings and Bliss, 1959) and on nitrogen cycling (Fisk et al., 1998) have been well demonstrated in different alpine sites.

An assessment of δ^13^C in forb and grass species from similar elevations (4100–4700 m) in New Guinea, the Venezuelan Andes, Mt. Kenya, and the northwestern Argentinean Andes, showed very similar δ^13^C values despite a decline in precipitation from >3000 mm a^-1^ (δ^13^C: -25.9‰) to 300 mm a^-1^ (δ^13^C: -26.3‰), thus revealing no differentiation by water relations (as captured by ^13^C signals) within such high elevation habitats (Körner et al., 1991). Additionally, δ^13^C in herbaceous plants along sharp moisture gradients in an Andean semi-desert in northwestern Argentina at 4200 m elevation exhibited no significant change from moist to dry sites (Körner, 2003). Such data suggest that stomatal responses to drought did not significantly affect δ^13^C even in alpine semi-deserts, presumably because wide plant spacing is balancing reduced precipitation, so that physiological responses to water availability did not restrict CO_2_ uptake per unit leaf area. Different species sampled from one common location were found to retain the δ^13^C signal they exhibit at separate locations, suggesting a strong genotypic influence on δ^13^C (Körner et al., 1991).

Similarly, it was found that foliar δ^15^N signals in co-occurring species varied with a constant rank across sites of varying N availability in a dry meadow community in the Rocky Mountains, suggesting a fixed partitioning of soil N and N utilization among these alpine plant species, regardless of habitats (Miller and Bowman, 2002). These findings suggest that δ^15^N signals have a strong genotypic component as well. Since foliar δ^15^N in non-N_2_-fixing species is tracking isotopic ratios of soil resources, several biological processes, such as mycorrhizal symbioses (Schmidt and Stewart, 2003) or horizontal transfer by mycorrhiza from N_2_-fixing species (Schulze et al., 1991) can influence ^15^N signals. It is well documented that non-N_2_-fixing plants may obtain N from N_2_-fixing plants via mycorrhizal connections and/or other ways (Johansen et al., 1993). The strong influence of N_2_-fixing plants on adjacent non-N_2_-fixing plants was evidenced recently for subtropical savannas in southern Texas (Bai et al., 2009) and steppe ecosystems in northern Mongolia (Casper et al., 2012).

Very cold environments do not preclude symbiotic N_2_-fixation, and so far, all legumes tested at high elevation were found to perform symbiotic N_2_-fixation (Wojciechowski and Heimbrook, 1984; Arnone, 1999; Jocot et al., 2000; Körner, 2003). This also holds for subarctic environments (Chapin et al., 1992). In fact, the relative contribution of symbiotically fixed N_2_ may even increase with elevation. Bowman et al. (1996) reported three species of *Trifolium* (Fabaceae) obtained a high proportion of their N from N_2_ fixation and played an important role in N cycling in the alpine site of Niwot Ridge in the Rocky Mountains. There are also other symbiotic N_2_ fixing associations in alpine vegetation, for example, actinomycorrhizal ones by *Dryas* species and subalpine *Alnus* (Körner, 2003; Hiltbrunner et al., 2014).

The primary objective of this study was to separate potential phenotypic (environment induced) from genotypic (phylogenetic) causes of carbon and nitrogen isotopic composition of plant species at high elevations. We (1) hypothesized that the variation in isotope discrimination is largely genotypic, given the supposed uniformity of the physical environmental conditions. Given the overall humid conditions in the test region (annual precipitation sums average at *c.* 1900 mm, Inauen et al., 2013) and the high elevation (*c.* 2430–2500 m), we (2) hypothesized that the balance of carbon and water relations as expressed in δ^13^C will result in a narrow signal range, at high absolute signals compared to low elevation, due to the 25% reduced atmospheric pressure. Based on previous studies, we (3) expected to find substantial differences in δ^15^N between legumes and non-legumes, and between early and late successional plant communities, but little additional variation within those categories, given the otherwise common life conditions. In addition, we (4) expected a strong influence of legumes on the δ^15^N signals of their non-legume neighbors. Finally, we had a methodological aim: we wanted to identify variation (and its causes) that may also influence the outcome of random sampling of isotope signals such as for instance from herbaria, where no information on site conditions, neighborhood, replication and often no choice in tissue is given. We, thus, systematically explored the influence of tissue type, variation among individuals sampled from the very same location, variation across species in interaction with different sites, community means for different community types, bulk biomass, and soil organic matter (SOM).

Hence, as a methodological task, this field study was also designed to provide some guidance on how to treat and interpret the variation in isotopic signals to be expected in studies based on archive material from alpine terrain. Isotopic signals may vary with the type and age of plant tissue sampled (Yoneyama and Ohtani, 1983; Högberg, 1997; Evans, 2001). Therefore, we explored organ specificity of isotopic signals in both C and N in alpine taxa from our test region. This may also contribute to the understanding of the causes of signal variations, that is, the responsible metabolic processes during dry matter allocation (Dawson et al., 2002).

## Materials and Methods

### Sampling Sites

Samples were collected in the vicinity of the ALPFOR Research Station (http://pages.unibas.ch/botschoen/alpfor/) at Furka pass (Swiss Central Alps) in locations between 2430 and 2500 m a.s.l. in the upper Reuss catchment (46°34′N, 08°25′E) in July 2012. In 2012, snowmelt started in early to mid June and the season ended with plant senescence between early and mid September.

The mean temperatures in June, July, August, and September were 4.9, 7.7, 6.9, 3.6°C during the last 3 years, with ca. 7.3°C during July 2012 when this field survey was conducted; and mean soil temperatures in 10 cm depth are 4 K warmer than air temperature (unpublished data of the ALPFOR station, E. Hiltbrunner personal communication). Annual precipitation varies around 1900 mm, with ca. 500 mm falling during the growing season (mid June to mid September), always exceeding evapotranspiration (Inauen et al., 2013). Soils under the vegetation examined are acidic (pH 3.1–4.1; Supplementary Table S1), rich in organic material, and mostly deeply weathered, with a high water holding capacity (alpine brownearth; van den Bergh et al., 2013). The flora of this region is among the richest in the Alps (Hefel and Stöcklin, 2010). More than half of the Swiss alpine angiosperm plant species (301 species) occur within ca. 1 km around the research station as a result of mixed geology, including both, carbonate-rich and siliceous bedrock.

### Plant, Soil and Biomass Sampling

In this project we adopted a ‘quasi-common-garden’ approach, by sampling the same species or individuals of the same species from homogenous sampling plots at different positions (horizontal transect of ca. 2 km) in an alpine landscape. This way, we can exclude significant environmental variation within a given sampling plot, cover a spectrum of different plant communities (including the same target species) at otherwise minor climatic and moisture contrasts among plots, and account for potential genotypic variation and legume-neigbourhood effects. In contrast to classical common gardens, the plants sampled were not transplanted but had a long history of *in situ* interaction with other plants, soil, and microbes (mycorrhizae), while belonging to the same metapopulation.

**FIGURE 1:**
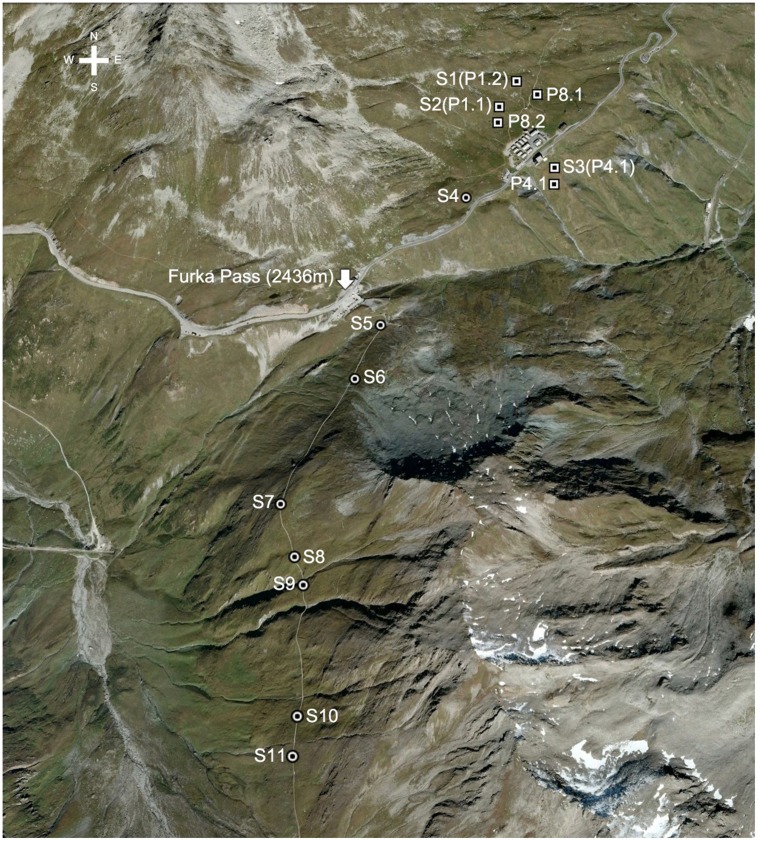
**Location of sampling sites (S, circles) near Furka Pass, Swiss Central Alps at 2430–2500 m and six permanent plots (P, squares) near the ALPFOR research station [adapted from Google Earth (version 7.1.2.2041)]**.

Samples were largely collected next to six permanent plots maintained by the ALPFOR research station (**Figure 1**), three types of which were chosen that encompass typical plant communities such as *Nardus*-rich heathland (P1.1 and P1.2), *Festuca violacea* meadows (P4.1 and P4.2) and snowbed communities (P8.1 and P8.2; Supplementary Table S1). In each of these sampling locations, leaf samples from different species, soil samples from different soil depths and samples of different types of plant tissue were collected (see below).

In addition to the plot-based sampling, 4–6 common species, i.e., one sedge (*Carex sempervirens*), one rush (*Luzula lutea*), one legume (*Trifolium alpinum*), two forbs (*Geum montanum*, *Homogyne alpina*), and one grass (*Poa alpina*) were collected from 6 to 8 additional sites along a ca. 2 km transect across varied alpine terrain in the same region (locations S1–S11, **Figure 1**), including three of the permanent plot locations (P1.1, P1.2, and P4.1; Supplementary Table S1). We arrived at 8–11 sites that had 4–6 of the species in common.

For foliar sampling, 3–5 healthy and green leaves from each of 3–5 (mostly 5) individuals per plant species were collected from a sampling location. In general, the species sampled were representative and important for each studied plant community (Supplementary Table S2). To minimize the possible impact of rhizobium-fixed N_2_ on foliar isotopic signatures, samples were collected at least 1 m away from legumes (mostly *T. alpinum* and rarely *T. thalii*). In order to investigate the impact of N_2_-fixing species on foliar ^15^N of adjacent non-N_2_-fixing species, leaves of three individuals of each of 6–8 non-N_2_-fixing species were collected in plots P1.1 and P1.2, co-occurring near (<10 cm) a patch of one common legume (*T. alpinum*). Except when we studied individuals, we pooled leaves for each species from each sampling location (foliage from 5 different individuals).

For bulk plant dry matter and soil samples, two subplots (25 × 25 cm) were used at the edge of each of the permanent plots. On each subplot, plants were clipped to 1 cm above ground, leaving the bottom moss layer untouched. Above-ground dry matter was then sorted into live (biomass), standing dead (necromass) and litter fractions. From the same subplot, a central soil core was extracted down to 30 cm depth using a 2.5 cm diameter metal corer. For each soil profile, segments at depths down to 2.5, 5, 10, 15, 20, 25, and 30 cm were bagged separately. The soil samples were sieved (<1 mm) after roots had been removed.

### Sampling of Different Organs within One Species

To assess the C and N isotopic compositions of different organs within a species, we have chosen four common species in the study region (i.e., *C. foetida*, *C. sempervirens*, *Nardus stricta,* and *T. alpinum*) and collected three individuals from each species close to plot P8.2. For each plant individual, different organs including terminal (older) and lower (younger) parts of green leaves (in case of *T. alpinum*, these were leaf petiole and leaf blade instead), dead leaves, flowering stalks, fruit heads, seeds and roots were collected.

### ^13^C and ^15^N Analysis

Except when we studied different organs or bulk biomass, the study was based on green leaves. All samples were dried at 80°C. The oven dry material was ground with a steel ball mill. Samples of 2–3 mg were then weighed into tin capsule. Dried and sieved (<1 mm) soil samples were ground manually with a mortar, and 4–5 mg was weighed into tin capsule.

The isotopic signature (^13^C and^15^N) of plant and soil materials were determined using an isotope ratio mass spectrometer (Delta S, Finnigan Mat, Bremen). Isotope ratios were calculated as:

$$\delta {[}^{13}C{,}^{\mathrm{15}}N]\mathrm{samples}=(R_{\mathrm{sample}}{\mathrm{/R}}_{\mathrm{standard}}-1)\;\times \;1,000$$

where R_sample_ and R_standard_ are the ^13^C/^12^C or ^15^N/^14^N molar abundance ratios of samples, with the internationally accepted standard (V-PDB) and the free atmosphere (^15^N), serving as references.

### Data Analysis

Differences in δ^13^C and δ^15^N among different organs within one plant species were examined using one-way ANOVA. Differences in δ^13^C and δ^15^N values between any two organs were examined using *post hoc* multiple comparison (LSD). Differences in δ^13^C and δ^15^N among species co-occurring in P1.1 and P1.2 in the *Nardus* heathland were examined using one-way ANOVA. Differences among mean values of foliar δ^13^C and δ^15^N of species in Cyperaceae (*C. sempervirens*, *C. curvula*), Juncaceae (*L. lutea*) and Fabaceae (*T. alpinum*) and species of other families co-occurring in P1.1 and P1.2 were examined using the Independent Samples Test. Difference in mean amplitude of δ^13^C and δ^15^N values for all examined species between P1.1 and P1.2 was examined using the Independent Samples Test. Difference in mean values of foliar δ^13^C and δ^15^N for the sampling sites at the six permanent plots, and among the 8 or 11 sites sampled along the transect, were examined using one-way ANOVA. The difference in foliar δ^13^C and δ^15^N between any two sites of the transect was examined using *post hoc* multiple comparison (LSD). For comparing differences in values of foliar δ^13^C and δ^15^N of non-N_2_-fixing species near and away from legumes (*T. alpinum*), data of foliar δ^15^N and δ^13^C in three individuals of each non-N_2_-fixing species was averaged for both positions and tested using the Independent Samples Test. Data for bulk community dry matter and each depth of the soil profile from different permanent plots but belonging to the same type of plant community were combined because signals between the two samples within a plot varied similarly than among plots yielding four replicates per habitat type. Then differences in bulk community dry matter were examined across the three types of communities using one-way ANOVA. Differences in δ^13^C and δ^15^N values of bulk green biomass and means for all species studied individually either with or without legumes in each plant community were examined using one-way ANOVA and *post hoc* multiple comparison (LSD). Differences in soil δ^13^C and δ^15^N were examined across the three plant community types and the different soil depths using the general linear model (GLM) procedure. These analyses were done using the SPSS package (Version 20, SPSS, Chicago).

## Results

### Intra-Plant (Tissue) Variation in δ^13^C and δ^15^N

**FIGURE 2:**
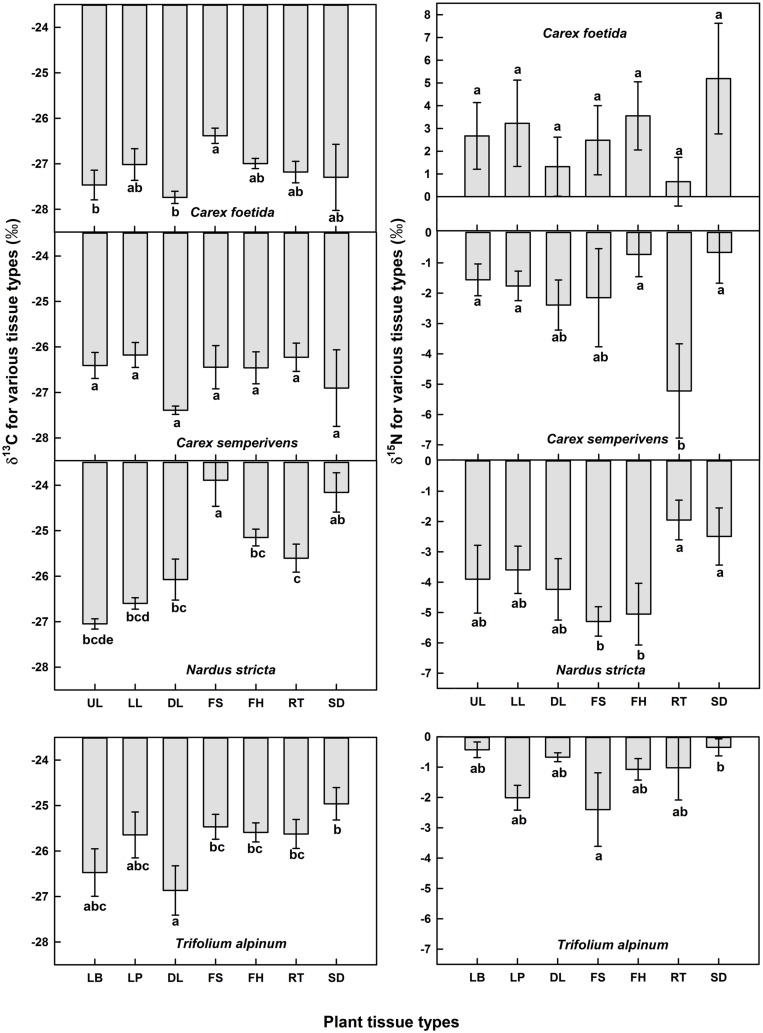
**Means of δ^13^C and δ^15^N signals in different organs in *Carex foetida*, *C. semperivens, Nardus stricta*, and *Trifolium alpinum* (mean ± SE, *n* = 3 individuals).** UL, upper leaf parts; LL, lower leaf parts; DL, dead leaves; FS, flowering stalks; FH, fruit heads; RT, roots; SD, seeds. For *T. alpinum*, LB, leaf blades; LP, leaf petioles. Bars with different letters indicate significantly different means (*p* < 0.05, pairwise *post hoc t-test*).

Overall, no statistically significant organ effect on δ^13^C was found in *C. foetida* (*p* = 0.26) and *C. sempervirens* (*p* = 0.48), although, some *post hoc* pairwise tests for individual organ types showed a difference in *C. foetida* (**Figure 2**). However, the organ effect on δ^13^C was significant in *N. stricta* (*p* < 0.001), and marginally significant in *T. alpinum* (*p* = 0.07), with fruits, seeds, roots, and flowering stalks generally less negative than green foliage (**Figure 2**). No statistically significant organ effect in δ^15^N was found in the four studied plant species, but again, pairwise tests revealed some significant contrasts (**Figure 2**). For δ^15^N, there was a trend for roots being different from the rest, but with no clear direction (and no such trend in *Trifolium*). The range among different means for different organs in the same species varied from 0.5 to 2.5‰ in δ^13^C, and from 2 to 4.5‰ in δ^15^N, hence can be substantial, and for each species, the difference in δ^15^N between green leaves and other organs was wider than in δ^13^C (**Figure 2**). In the following we will report green leaf data only.

### Local Intra-Specific (Individualistic) Variation in Foliar δ^13^C and δ^15^N

**FIGURE 3:**
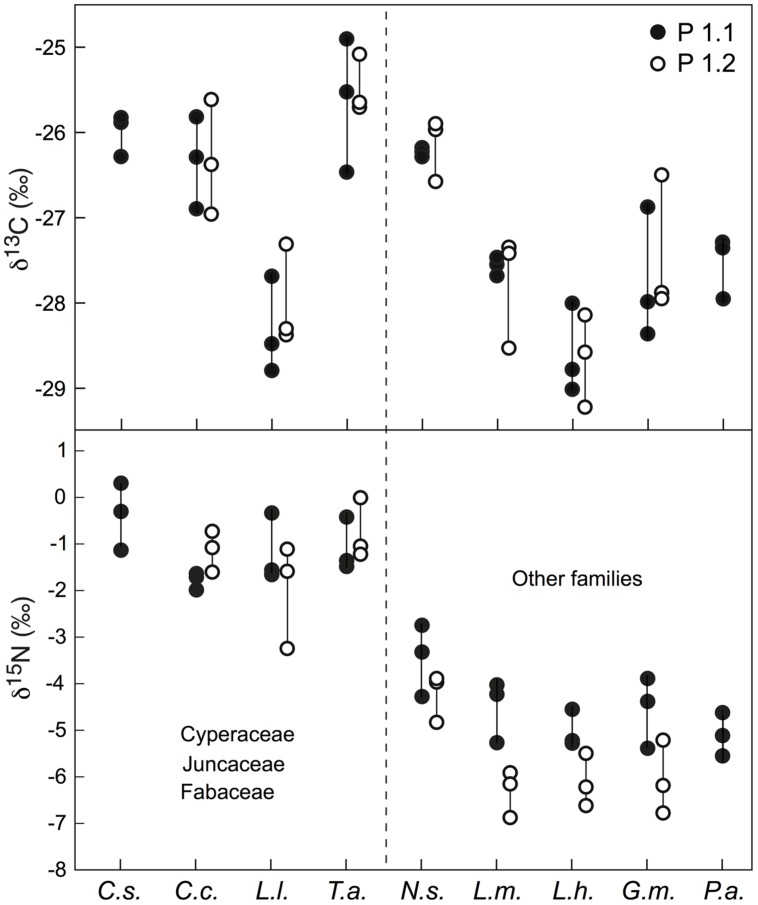
**Intra-specific variation in δ^13^C and δ^15^N among three individuals growing next to each other for nine or seven species sampled at two sites (P1.1 and P1.2).** Each point represents a mixed signal from several leaves for one individual. The species effect across this set of species (based on values for three individuals per species) was significant in both plots at *p* < 0.01 for both δ^13^C and δ^15^N. *Cs*, *Carex sempervirens*; *Cc*, *Carex curvula*; *Ll*, *Luzula lutea*; *Ta*, *Trifolium alpinum*; *Ns*, *Nardus stricta*; *Lm*, *Ligusticum mutellina*; *Lh*, *Leontodon helveticus*; *Gm*, *Geum montanum*; *Pa*, *Potentilla aurea*.

In green leaves of 7–9 species, there was a consistent intra-specific variation in δ^13^C of ca. 1.0‰ among three individuals grown next to each other (**Figure 3**). That variation was consistent across two different test sites (P1.1 and P1.2) in the *Nardus* heathland. The single highest deviation in δ^13^C signals among the three tested individuals per location was 1.6 and 1.5‰, with a mean range size (amplitude) for all examined species of 1.1 and 0.9‰ for P1.1 and P1.2, respectively. In δ^15^N signals, consistent across sites, one individual may differ from another one sampled at the same location (separated by a few centimeters) by up to 1.5 and 2.1‰, with a mean amplitude among three individuals of 1.1 and 1.3‰ for P1.1 and P1.2, respectively (**Figure 3**). However, no significant difference was found in mean amplitude of δ^13^C and δ^15^N values for all examined species between P1.1 and P1.2.

### Intra-Specific Variation of δ^13^C and δ^15^N Across Different Sites

**FIGURE 4:**
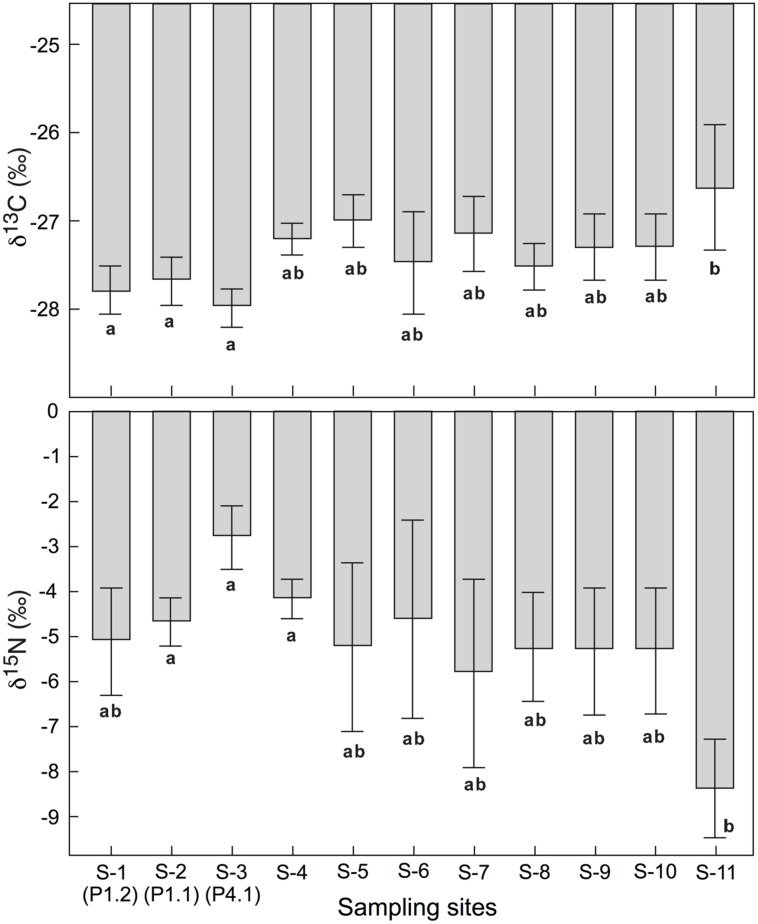
**Means of foliar δ^13^C and δ^15^N for four co-occurring alpine plant species in all 11 sites (mean ± SE).** There was no overall site effect across all sites. But means for some individual sites differed (different letters indicate significant differences at *p* < 0.05).

No significant difference in foliar δ^13^C and δ^15^N was found across all 11 sites where the four species *P. alpina*, *Geum montanum*, *Homogyne alpina,* and *L. lutea* jointly occurred (mean for five individuals per site; **Figure 4**). When site effects were tested site by site, foliar δ^13^C at site S11 (a steep meadow close to a glacier fore-field) showed significantly (or marginally significantly) less negative signals than site S1, S2, and S3 (**Figure 4**). Additionally, foliar δ^13^C at site S5 were marginally less negative than foliar δ^13^C at site S3. Also foliar δ^15^N made site S11 special, as leaves had significantly (marginally significantly) more negative δ^15^N than these collected in sites S2, S3, S4, and S6 (**Figure 4**). Similarly, no overall differences in species-specific foliar δ^13^C and δ^15^N were found across those eight sites where the same six species co-occurred (i.e., *P. alpina*, *G. montanum*, *H. alpinum*, *L. lutea*, *T. alpinum*, *C. semperivens*). Tested site-by-site, similar differences emerged as for the four-species-comparison reported above.

**FIGURE 5:**
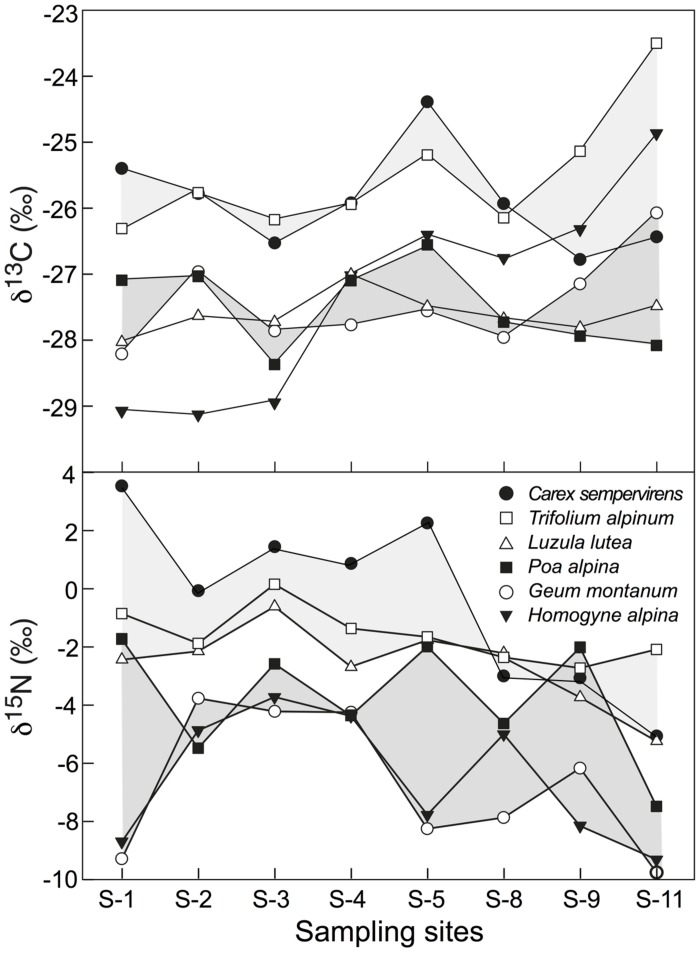
**Inter-specific variation in foliar δ^13^C and δ^15^N in six alpine species common to eight sites spread over a 2 km transect at c. 2430–2500 m elevation (**Figure 1**), with each point representing the pooled signal for several leaves sampled from 5 individuals per species and site.** Shades are visualizing distinct high or low signal groups, with δ^13^C in *Homogyne* not matching any group.

In δ^13^C, *Trifolium* and *Carex* were consistently less negative than *Geum*, *Poa,* and *Luzula*, with *Homogyne*, in contrast, changing from -29‰ at S1 to -25‰ at S11 (**Figure 5**). In δ^15^N, *Trifolium*, *Carex,* and *Luzula* were consistently less negative than *Geum*, *Poa,* and *Homogyne*. However, the difference from one location to the other may reach 2.7‰ (3.8‰ in *Homogyne*) in these species, except for *Trifolium* and *Luzula,* which hardly varied (**Figure 5**). Disregarding *Homogyne*, the mean site to site variation in the remaining four species was >0.5 and <1‰, with most of the variance produced by site 11 (mean ± SD of five individuals for the eight sites for each species, *Carex* -0.4 ± 3.0‰; *Trifolium* 1.6 ± 0.9‰; *Luzula* -2.6 ± 1.4‰; *Poa* -3.8 ± 2.1‰; *Geum* -6.7 ± 2.4‰; *Homogyne* -6.5 ± 2.2‰). Similarly, four out of the seven species co-occurring in both sampling plots in the *Nardus* heathland showed significant (or marginally significant) differences in δ^15^N between P1.1 and P1.2 (*C. curvula*: *t* = -2.325, *p* = 0.081; *Ligusticum mutellina*: *t* = 3.373, *p* < 0.05; *Leontodon helveticus*: *t* = 2.724, *p* = 0.053; *G. montanum*: *t* = 2.367, *p* = 0.077). However, no difference was found in foliar δ^13^C between P1.1 and P1.2. Therefore, the intra-specific amplitude of changes between locations (P1.1 and P1.2) was wider for foliar δ^15^N than for foliar δ^13^C, in particular for species classified as ‘other families’ (**Figure 3**).

### Inter-Specific Variation in Foliar δ^13^C and δ^15^N

**FIGURE 6:**
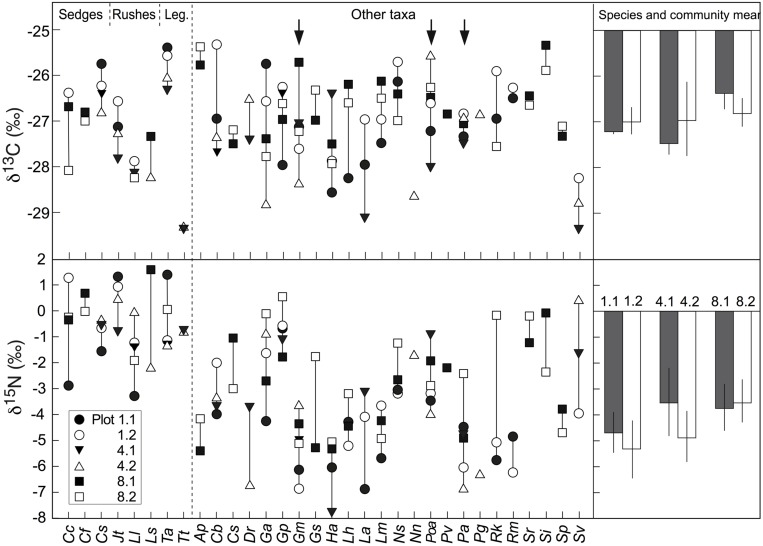
**Left: interspecific variation in the natural abundance of δ^13^C and δ^15^N signals in alpine plant species from six different plant communities (five individuals per species and plot).** Arrows indicate three species that occurred at all six permanent plots. Sedges include: *Cc, Carex curvula*; *Cf, Carex foetida* and *Cs, Carex sempervirens*; Rushes include: *Jt, Juncus trifidus*; *Ll; Luzula lutea* and *LS*, *Luzula sudetica*; Legumes include: *Ta, Trifolium alpinum* and *Tt, Trifolium thalii* (see Supplementary Table S2 for the full name of the rest plant species collected in each plot); Right: means of δ^13^C and δ^15^N in three species occurred at all six permanent plots (mean ± SE, *n* = 3 species). Bars with different color means different plots. None of the means of δ^13^C and δ^15^N differed significantly among the six plots (*p* > 0.6).

Overall, the δ^13^C values of co-occurring species in the three alpine plant communities tested varied from -25.5‰ to -29.5‰, with mean signal ranges per community of around 3.2, 3.8, and 4.4‰ for the *Nardus*-rich heathland (plots P1.1 and P1.2), the *F. violacea* meadow (plots P4.1 and P4.2) and the snow-bed community (plot P8.1 and P8.2), respectively (**Figure 6**). Variation in δ^15^N within each community was even wider (**Figure 6**). In alpine *Nardus*-rich heathland, the δ^15^N values ranged from 1.4‰ (*T. alpinum*) to -6.9‰ (*Leucanthemum alpina*), a range of 8.3‰. After excluding data of *Trifolium*, sedges and rushes, the δ^15^N value of co-occurring species in this plant community still ranged from about -6.9 to -0.6‰. For the *F. violacea* rich meadows, the amplitude covered a range in δ^15^N of 7.3‰ irrespective of whether data of *Trifolium*, sedges and rushes were excluded or not. For the snow-bed community, the amplitude was similar at 7‰ (6‰ when data of *Trifolium*, sedges and rushes were excluded). The δ^15^N values of most forbs and grasses were lower (more negative) and varied more widely than those in sedges (*C. curvula, C. foetida, C. sempervirens*), rushes (*Juncus trifidus, L. lutea*), and legumes (*T. alpinum*, *T. thalii*; **Figure 6**). Similarly, significant differences in δ^13^C and δ^15^N were found among species occurring in both *Nardus* heathland plots (*p* < 0.001 for both isotopes), with higher mean values of δ^13^C and δ^15^N in Cyperaceae (*C. sempervirens*, *C. curvula*), Juncaceae (*L. lutea*), and Fabaceae (*T. alpinum*) than species in other families (*p* < 0.001 for δ^13^C both plots; *p* < 0.001 in P1.1 and *p* = 0.086 in P1.2 for δ^15^N; **Figure 3**).

Of the forbs, three species (*G. montanum*, *P. alpina*, *Potentilla aurea*) were studied in all six permanent plots and did not reveal any spatial pattern of δ^13^C and δ^15^N, but as a group, the three species were significantly different in their δ^15^N signature from either *Trifolium* or sedges/rushes and the combined legume/sedge/rush signal obtained from the same sampling locations. No significant differences in δ^13^C and δ^15^N were observed across all six plots and communities co-composed by these three species (**Figure 6**).

The foliar δ^15^N and %N were not correlated (*r*^2^ = 0.011, *p* = 0.297) when compared across all sites and species. However, these two parameters were marginally correlated when data of *Trifolium*, sedges and rushes were removed from the analysis (*r*^2^ = 0.050, *p* = 0.051).

### Effect of Legume Neighborhood on Foliar δ^15^N

δ^15^N for 6–8 species either growing next to or away from *T. alpinum* showed no significant difference (-3.24‰ with and -3.95‰ without *Trifolium* at plot P1.1 and -4.30‰ with and -3.18‰ without *Trifolium* at plot P1.2; Supplementary Table S3). The results did not differ when δ^15^N values of sedges (*C. curvula* and *C. sempervirens*) and the rush (*L. lutea*) were removed from the data set (Supplementary Table S3).

### Signals in Bulk Plant Dry Matter in Different Plant Communities

**FIGURE 7:**
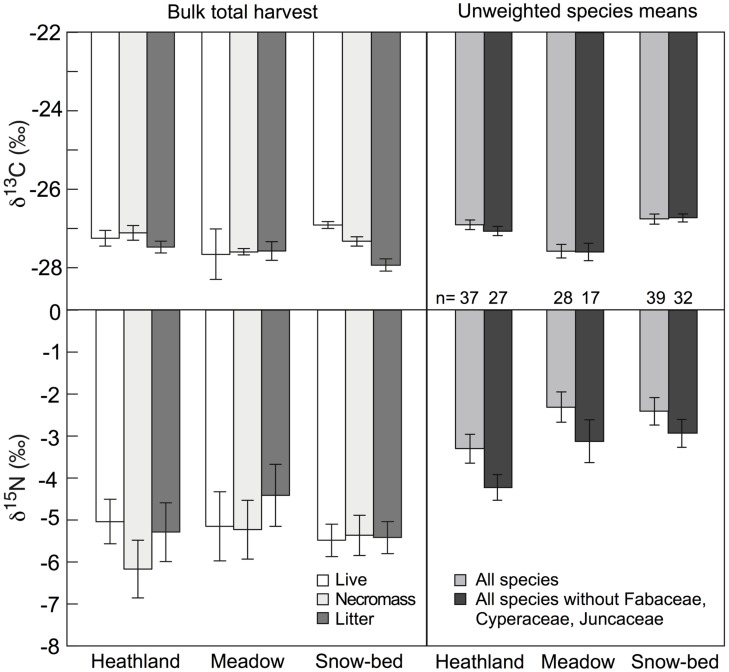
**Left: means of δ^13^C and δ^15^N signals in different types of bulk plant dry matter fractions in *Nardus* heath, *Festuca* meadow, and snow-bed communities (mean ± SE, *n* = 4 harvested sub-plots per community type).** None of the means of δ^13^C (live: *F* = 0.9, *p* = 0.427; necromass: *F* = 3.1, *p* = 0.095; litter; *F* = 1.6, *p* = 0.246) and δ^15^N (live: *F* = 0.1, *p* = 0.866; necromass: *F* = 0.6, *p* = 0.547; litter: *F* = 0.7, *P* = 0.5) differed significantly from each other. Right: means of δ^13^C and δ^15^N in green leaves for all species studied individually (see **Figure 6**), with (gray) and without (black) Fabaceae, Cyperaceae, and Juncaceae (the individual signal is for one species, so the replicated unit is the species in this case).

No significant difference was found in δ^13^C and δ^15^N between live (green) plant material (biomass), necromass and litter across the three studied plant communities (**Figure 7**). When samples in bulk green biomass were compared with the unweighted (disregarding species abundance) mean of the species sampled in each community for δ^13^C and δ^15^N (**Figure 6**), either with or without legumes in each studied community, no significant difference in δ^13^C among communities was found (**Figure 7**). However, δ^15^N in bulk biomass was 2–3‰ more negative than the corresponding means calculated from the species data (**Figure 7**), indicating that species with more negative δ^15^N contributed over-proportionally to total biomass (e.g., grasses and snowbed herbs, all operating at quite negative δ^15^N).

### Soils Under Different Plant Communities

**FIGURE 8:**
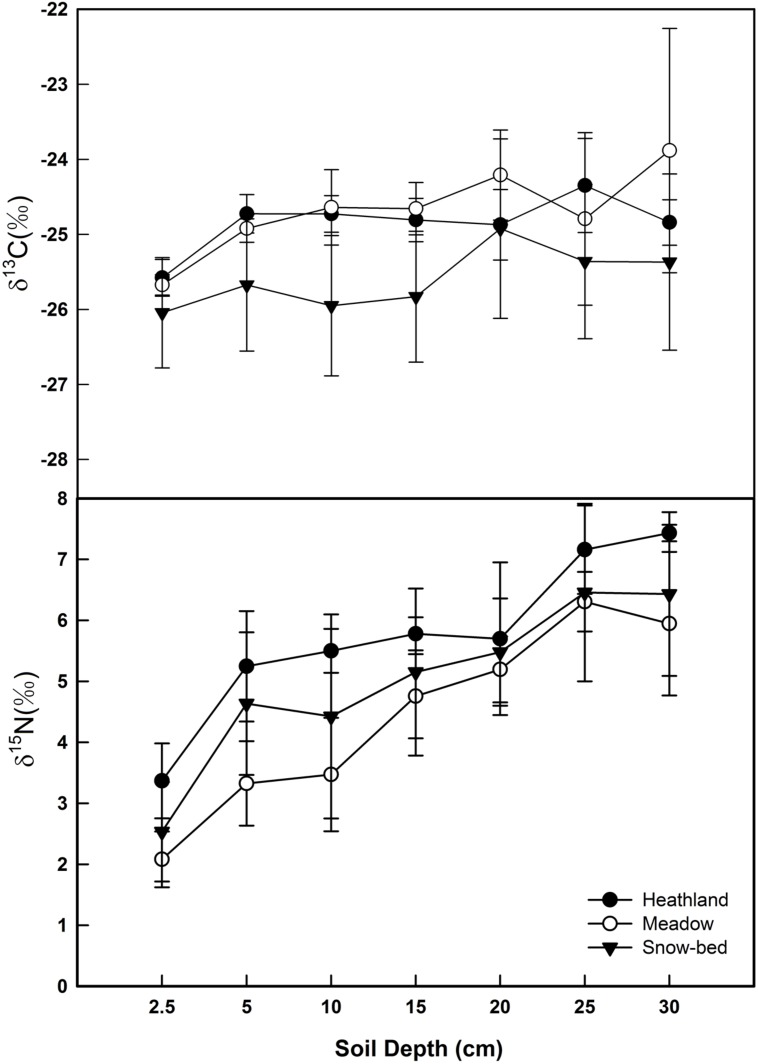
**Soil profiles of δ^13^C and δ^15^N in the three alpine plant communities (mean ± SE, *n* = 4 soil samples per community type).** While no significant effects of soil depth on δ^13^C, δ^15^N increased significantly with soil depth (*p* < 0.001).

The δ^13^C signal in soil organic carbon showed significantly higher (less negative) values than the bulk litter fraction in the heathland (*p* < 0.001), the meadow (*p* = 0.001) and the snowbed (*p* = 0.017) communities, thus did not mirror the mean litter signal. There was no significant effect of soil depth on δ^13^C (**Figure 8**). Also soil δ^15^N was much higher than the values for most plant species’ tissue and for bulk dry matter of each plot studied. In contrast to δ^13^C, δ^15^N increased significantly with soil depth at all sites (**Figure 8**, with a vertical range of about 4‰, offering a wide spectrum of isotopically different soil N sources for different plant species). Overall, no difference in soil δ^15^N was found across the three types of plant communities. However, the soil δ^15^N in the heath community (plots P1.1 and P1.2) was marginally higher than that in the meadow community (plot P4.1 and P4.2; *p* = 0.051).

## Discussion

To our knowledge, no systematic study has been conducted in alpine terrain to separate environmental from phylogenetic C and N stable isotope signals of plant species. In order to explain phenotypic signals, we examined δ^13^C and δ^15^N in plants in three different plant communities and in a number of different habitats related to topography in the central Alps. By examining δ^13^C and δ^15^N values in different tissue types as well as bulk community biomass, we were able to illustrate the full spectrum of sources of isotopic variation. Since we conducted this survey in an alpine landscape on well developed soils with no biologically relevant variation in moisture availability, the substantial signal variation at all organizational levels, except the ecosystem/community level, came at a surprise.

Overall, the mean intra-species variation in δ^13^C of ca. 1.1‰ (extreme 1.6‰) and the mean variation among tissue types of ca. 1.2‰ (extreme 3.2‰), together exert a largely unexplained ‘noise’ that calls for broad and highly replicated sampling for obtaining a species-specific and/or habitat-specific, representative signal. The intraspecific variation in δ^15^N was almost twice as large as that in δ^13^C. The δ^13^C and δ^15^N signals in the species examined, varied strongly among all plant families, and inconsistently with sampling location, but the rank order among species was retained across sites with only few exceptions (i.e., was predominantly genotypic). In the following we will discuss the intra- and inter-specific variation in isotope signals in this alpine environment.

### Intra-Plant, Tissue-Specific Signals

At intra-plant level, two (*Nardus stricta* and *Trifolium alpinum*) out of the four species explored for all major tissue (organ) types showed significant variation in δ^13^C values across different organs (mostly around 1‰). Substantial variation in δ^13^C in different parts of plants was reported in previous studies (O’Leary, 1981; Yoneyama and Ohtani, 1983; Saranga et al., 1999; Chevillat et al., 2005). Hence, uncontrolled tissue type would add up to 1.0–3.0‰ of deviation from means for different organs in *C. sempervirens* and *N. stricta*. For *N. stricta* and *T. alpinum*, less negative δ^13^C values in stalks, seeds, fruit heads and roots compared to green leaves match the trends compiled for non-leaf tissue (O’Leary, 1981). The enrichment of δ^13^C in roots relative to foliage suggests an isotopic fractionation between foliage and roots (Hobbie et al., 2002). In legumes, CO_2_ fixation driven by PEP carboxylase in active nodules, roots, seeds, and other tissue (Coker and Schubert, 1981) may lead to the less negative δ^13^C values. However, we have no good explanation for the δ^13^C enrichment of autotrophic *versus* heterotrophic tissues in *N. stricta.* Perhaps, this is related to the seasonal timing of the respective C uptake or to specific turnover of reserves. Similarly, δ^15^N varied substantially among the tissues of the tested plant individuals, mostly by >2‰. Such tissue-specific differences in δ^15^N and the often diverging signals in roots have been reported before by Evans (2001), hence, alpine species make no exception. ^15^N discrimination during reallocation of N is one plausible explanation (Evans, 2001).

### Intra-Specific, Individualistic Signals

The data we presented fall in two categories: individuals sampled at the same spot, often not further apart than a few centimeters, *versus* groups (means) of five individuals sampled along a several km transect with locations separated by at least 50 m distance. For differences in δ^13^C between individuals growing next to each other, we have no other explanation than individualistic (genotypic) differences in gas exchange traits, given the consistently high soil moisture and the same climatic conditions. However, for δ^15^N, the remarkable intra-specific (individualistic) variability may reflect the heterogeneity of substrate and/or rooting patterns, or differences in mycorrhization (Craine et al., 2009). For intra-specific variation across different sites, mixed leaf samples from single plant individuals, growing next to each other may differ in δ^13^C by as much as different tissues on the same plant do (ca. 1‰) with the variation nearly twice as large in δ^15^N. Hence, the local plant to plant variation was nearly as high as that among the means for micro-populations of five individuals per site, across the 8–11, widely spaced sites (disregarding *Homogyne* that varies more with site). Possible causes for the twice as large variation in δ^15^N among these sites could be different N-availability, uptake of different N forms (i.e., NO_3_^-^ and NH_4_^+^) or mycorrhization (Miller and Bowman, 2002). In habitats where nitrogen is limiting, plants will acquire nitrogen largely from mycorrhizae. Mycorrhization is known to alter plant δ^15^N (Högberg, 1997; Evans, 2001; Craine et al., 2009).

The exceptional values obtained for the means of five individuals per species on site S11 (a steep mountain pasture) may be related to the frequent presence of sheep (personal observation), with significantly less negative δ^13^C and more negative δ^15^N than in all other sites. In montane riparian habitats in the Rocky Mountains, browsing of *Salix* by elk significantly increased leaf δ^13^C, perhaps due to the shift in root to shoot ratio (Alstad et al., 1999). In addition, browsing or grazing can also change plant δ^13^C by affecting per unit leaf area water loss (lower LAI but more intense gas exchange; Kielland and Bryant, 1998). Since carbohydrate reserves tend to be enriched in ^13^C relative to fresh photo assimilates, their allocation to with-season re-growth after grazing may have contributed to these signals. Also, better *N*-nutrition might increase the efficiency of C fixation. Similar to our signals, a grazed grassland community in the Yellowstone National Park showed reduced plant δ^15^N (isotopically lighter soil NO_3_^-^ may constitute a more important N resource for plants in grazed areas; Frank and Evans, 1997).

### Inter-Specific Variation

Inter-specific variation in δ^13^C (on top of intra-species and tissue-specific variation as discussed above) is very large, causing the actual signals obtained for a given plant community and elevation to strongly depend on the species included, sample replication per species and tissue type. For archive-based assessments of environmental signals for a given type of ecosystem, this requires a multi-species approach (presumably > 30 species) to overcome the random nature of such vouchers with regard to individuals, tissue quality and potential micro-habitat effects.

In this study, the difference in foliar δ^13^C among co-occurring plant species within each studied community falls in the 2–5‰ range in foliar δ^13^C for terrestrial plant communities composed of C3 species reported by O’Leary (1981). Given the high rainfall regime and well developed soils at our test sites, all plants were well supplied with moisture. Hence, the differences among species should largely reflect genotypic signals (Dawson et al., 2002). Several genotypic factors such as leaf mesophyll thickness and CO_2_ uptake efficiency have been related to foliar δ^13^C through their influence on factors such as leaf boundary layer resistance, diffusive conductance (i.e., stomatal and intercellular resistance) and mesophyll resistance to CO_2_ uptake (Körner et al., 1988, 1991; Geber and Dawson, 1990; Vitousek et al., 1990).

It is interesting that foliar δ^13^C in the legume *T. alpinum* and the sedge *C. sempervirens* showed consistently higher (less negative) values than the other four species tested across eight sites. Legumes can perform dark fixation of CO_2_ in root nodules by PEP carboxylase (Coker and Schubert, 1981), which hardly discriminates against ^13^C (Estep et al., 1978), thus leading to less negative δ^13^C signal in legumes (Yoneyama and Ohtani, 1983). The high nitrogen concentration may also facilitate higher carboxylation capacity relative to stomatal conductance and may, thus, further contribute to the less negative δ^13^C signal in *Trifolium*. The special position of Cyperaceae and Juncaceae in terms of δ^13^C among other taxa is difficult to explain. Körner et al. (1991) suggested that species with large below-ground biomass, such as *Ranunculus glacialis* and *Oxyria digyna* showed less discrimination than those with very small below ground components like *Arabis alpina*. *C. sempervirens* is among the most strongly and deeply rooted species of the five studied species and had less negative δ^13^C values than other species (*L. uzula lutea*, *P. alpina,* and *G. montanum*). Again, this could be related to a greater contribution of ^13^C enriched carbohydrate reserves to the rapid formation of new leaves after snow melt. Forthcoming study may now be needed to examine the seasonal course of carbon isotope discrimination in alpine plant species to evaluate changes in gas exchange process in response to biotic and abiotic factors.

At species level, we also observed a wide interspecific variation in foliar δ^15^N (ca. 7–8‰) within each studied community. Similar ranges in foliar δ^15^N were reported for arctic tundra communities in Alaska and Swedish Lapland (Michelsen et al., 1996; Nadelhoffer et al., 1996), while foliar δ^15^N of species in temperate and tropical regions typically differ by less than 5‰ for a given site (Garten, 1993; Nadelhoffer and Fry, 1994). According to Craine et al. (2009), the variation in foliar δ^15^N in cold ecosystems with mean annual temperatures of around 0°C would be even wider (ca. 15‰) when foliar δ^15^N data for some coniferous species were included. In the Rocky Mountains of Colorado, Miller and Bowman (2002) explained the difference in δ^15^N by the species-specific ability to absorb NH_4_^+^, NO_3_^-^ or organic N (e.g., glycine). Low (very negative) foliar δ^15^N occurred in shallow rooted ericaceous species (*Vaccinium*, *Empetrum*, and *Ledum*), while high (less negative or even positive) values occurred in deeply rooted sedges (*Eriophorum* and *Carex*) in arctic tundra, where δ^15^N in soil was found to increase with soil depth (Nadelhoffer et al., 1996; Welker et al., 2003) as was found in our survey. Therefore, besides mycorrhization, the differences in rooting depth and/or age of organic N-sources may be another explanation for the great variation in foliar δ^15^N we found.

The rank order of foliar δ^15^N of six species that co-occur at one common site was largely maintained across sites. A similar consistent ranking of foliar δ^15^N among plant species and plant groups was reported for an alpine dry meadow and an arctic tundra ecosystem (Nadelhoffer et al., 1996; Miller and Bowman, 2002) suggesting persistent genetic differences with respect to N utilization of these species, regardless of overall N availability and site characteristics (Miller and Bowman, 2002). Legume species have generally higher δ^15^N value (close to zero) than non-N_2_-fixing plants, because of the small isotopic fractionation during fixation of atmospheric N_2_ by rhizobia (Rennie et al., 1978). Cyperaceae and Juncaceae always showed very high δ^15^N values as well. Species of these families seem to profit from their capacity of accessing very stable forms of ^15^N-enriched organic N in the soil (Körner, 2003). Surprisingly, Cyperaceae and Juncaceae do not differ from Fabaceae and, thus, these three families form a clearly distinct cluster of taxa separated from the rest of all other taxa.

For non-N_2_-fixing species, the correlation between foliar %N and δ^15^N was only marginally significant. Similar correlations were found at global to regional scales (Craine et al., 2009) including some low-N, primary successional sites in Hawaii (Vitousek et al., 1990), Zambia (Högberg and Alexander, 1995), and Alaska (Schulze et al., 1994; Hobbie et al., 2000). These correlations were suggested to reflect response to soil N availability, in either mycorrhizal N-uptake, a reduced fraction of mycorrhizal N transferred to plants or both (Hobbie et al., 2000). No such correlation was seen in N_2_-fixing plants, which presumably rely less on mycorrhizal derived N.

### Legume Effect on Non-Legumes

The presence of legumes may influence the availability and isotopic signature of N in the soil in their vicinity, producing δ^15^N signals in adjacent non-N_2_-fixing plants (Bai et al., 2009; Casper et al., 2012). In our study, the δ^15^N of *T. alpinum* was significantly higher than in non-N_2_-fixing plants (except for Cyperaceae and Juncaceae). Yet, we see no significant differences in δ^15^N between non-N_2_-fixing plants close to and away from N_2_-fixing *T. alpinum,* which is puzzling. We attribute this to two facts: (1) the unknown past location of legume individuals in these communities (random historical legume presence influencing N in SOM), and (2) the legume signal matches with that of the most abundant sedges, the neighborhood of which no species can avoid in this environment. Similarly, N_2_-fixing *Acacia tortilis* in savanna regions was found to exert no influence on adjacent non-N_2_-fixing plants (Handley et al., 1994), although the reasons might be different.

### Community Means and Soil Signals

Under natural non-alpine conditions, foliar δ^13^C was suggested to be largely related to soil moisture gradients (O’Leary, 1981), but the high elevation mosaic of life conditions does not exert such effects, not even in semi-arid mountains (see Introduction). Further, it is known that plants from alpine elevations usually have less negative δ^13^C than lowland taxa, largely a response to reduced atmospheric pressure (Körner et al., 1991; Zhu et al., 2010; Zhou et al., 2011), provided comparisons are not confounded by moisture stress at lower elevation. All the sampling sites of our study were open habitats without substantial gradients in elevation or moisture, which explains the lack of a significant site difference in δ^13^C signals at the community level.

The overall mean δ^13^C of -26.15‰ is slightly less negative as one would expect from the ca. 2500 m elevation a.s.l., a mean lowland signal of -28.8‰ (Körner et al., 1988) and a 1.2‰ mean change per 1000 m of elevation that emerged from earlier works. Assuming lowland reference data are for 100 m a.s.l. the expected drop in fractionation during leaf gas exchange to our mean site elevation by 2.9‰ would lead to a mean δ^13^C of -25.7‰. That is quite close to the -26.15‰, and thus, within the precision of such surveys. The elevational adjustment of leaf gas exchange to reduced partial pressure of CO_2_ is very species-specific (Körner and Diemer, 1987) and, thus, the species selected will influence the mean δ^13^C signal for a given elevation.

The less negative δ^13^C in SOM suggests that ‘lighter’ carbon was recycled faster, with the remaining SOM becoming δ^13^C-richer than the organic debris that entered the SOM pool. Similar increases in δ^13^C of SOM with soil depth relative to that of the litter layer were reported in forest ecosystem (Boström et al., 2007). The increased contribution of microbially derived C to SOM with depth was suggested to be the most likely mechanism accounting for this change (Ehleringer et al., 2000; Boström et al., 2007). This local shift in SOM δ^13^C is not in conflict with an overall correlation of soil and plant signals as was shown for an elevational gradient in Papua New Guinea (soil data by Bird et al., 1994; plant data in Körner, 2003).

A depth-related increase in ^15^N concentration in soils has been observed in forest, grassland and tundra ecosystems (Nadelhoffer and Fry, 1994; Nadelhoffer et al., 1996; Högberg, 1997). The discrimination against ^15^N during mineralization produces δ^15^N-enriched organic residues, and, unlike δ^13^C signals in SOM, these residues appear to constitute a greater fraction at greater soil depth (Nadelhoffer and Fry, 1994; Nadelhoffer et al., 1996; Högberg, 1997). We found that most plant species were depleted in ^15^N more than even the uppermost (0–2.5 cm) soils in all studied communities, matching observations from deciduous forest, coniferous temperate forest, alpine dry meadow and arctic tundra community (Garten, 1993; Nadelhoffer et al., 1996; Miller and Bowman, 2002). Compared to bulk soil, foliar litter and fine root litter are generally depleted in ^15^N, whereas sporocaps of mycorrhizal fungi are often enriched in ^15^N. Therefore, inputs of ^15^N-depleted root litter and ^15^N-enriched mycorrhizal fungi at depth could result in soil being substantially enriched in ^15^N relative to surface litter. Moreover, the common practice of sieving soil prior to sampling may preferentially remove root litter, potentially increasing the perceived contribution of fungal litter to total soil N and could therefore potentially influence the nitrogen isotope profile in soil (Hobbie et al., 2000). On the other hand, the partial conversion of nitrate during denitrification could strongly influence soil ^15^N by creating a pool of ^15^N-enriched nitrate that could be subsequently reassimilated by the microbial community, and ultimately increase soil δ^15^N (Hobbie et al., 2000). Therefore, the preferential uptake/microbial release of ^15^N-depleted inorganic N fractions and fractionation during internal N translocation have been offered as explanations (Högberg, 1997; Hobbie et al., 2000).

## Conclusion

We found a much wider spectrum of δ^13^C and δ^15^N signals in herbaceous and graminoid plant tissues than would be expected from the common (and harsh) environmental conditions the target plants species experience. The observed signal variation cannot or not obviously be attributed to the variation in growth conditions. The negligible influence of both, topography and the proximity of N_2_-fixing legumes, further suggest that δ^13^C and δ^15^N signals in the studied alpine plant communities varied for largely genotypic reasons and/or – in the case of ^15^N – for reasons related to species-specific plant-microbe/fungus interactions. We joining Högberg et al. (2005) in considering mycorrhizae as part of the autotrophic system, hence in the natural setting employed here, the term ‘genotypic’ includes species or genotype specific mycorrhization, particularly with regard to ^15^N signals. While the significant inter-specific differences of foliar δ^15^N within a community, most likely reflect access to different soil N sources, the variation in δ^13^C must reflect inherently different discrimination regimes (both physical and biochemical) in leaves during photosynthesis. These large and consistent inter-specific differences in stable isotope signatures among such herbaceous alpine plant species underline that elevational trends in stable isotope signals either need to be studied within a taxon [e.g., congeneric comparisons such as those by Zhu et al. (2010) and Zhou et al. (2011), or they must rely on a broad sample of species that covers the genetic signal diversity].

Sampling different tissue types will, on average, add at least another 1–2‰ of ‘noise’ (less in δ^13^C, more in δ^15^N), similar to the range of variation among individuals of the same species growing next to each other. Combined, our findings evidence that alpine plant species have evolved rather diverse means for acquiring and using CO_2_ and nitrogen. Moreover, the fairly consistent rank order of species in both, foliar ^13^C and ^15^N across different habitats, suggests that these traits are fixed and likely contribute to the functionality of alpine plant species diversity, very similar to what had been reported recently for sand dunes (Bermudez and Retuerto, 2014). At ecosystem scale, the high δ^15^N signal in the often dominant alpine Cyperaceae and Juncaceae (δ^15^N similar to legumes), does impose a significant isotopic fingerprint on the ecosystem N cycle, thus, masking isotopic legume neighborhood effects on non-N_2_-fixing taxa.

## Author Contributions

CK conceived and designed the work. YY performed work and analyzed the data. YY and CK wrote the manuscript. RS provided editorial advice on work and writing of the manuscript.

## Conflict of Interest Statement

The authors declare that the research was conducted in the absence of any commercial or financial relationships that could be construed as a potential conflict of interest.
