# Supplementary material for: Species specific and environment induced variation of δ13C and δ15N in alpine plants
Source: Front Plant Sci. 2015 Jun 5;6:423. doi: 10.3389/fpls.2015.00423 (PMC4456574; doi:10.3389/fpls.2015.00423)
Supplement: Supplementary file 1 [file Table_1.DOCX]

***Supplementary Material***

**Species specific and environment induced variation of δ13C and δ15N in alpine plants**

Yang Yang^1, 2^*, Rolf T. W. Siegwolf^3^, Christian Körner^2^

^1^ Key Laboratory for Plant Diversity and Biogeography of East Asia, Kunming Institute of Botany, Chinese Academy of Sciences, Kunming, Yunnan, China

^2^ Institute of Botany, University of Basel, Basel, Switzerland

^3^ Lab for Atmospheric Chemistry, Paul Scherrer Institute, Switzerland

**Correspondence**:

Dr. Yang Yang

Key Laboratory for Plant Diversity and Biogeography of East Asia, Kunming Institute of Botany, Chinese Academy of Sciences

Yunnan, Kunming, 650204, China.

[yangyang@mail.kib.ac.cn](mailto:yangyang@mail.kib.ac.cn)

**Supplementary Table 1.** Locations, elevations and soil characteristics of six permanent plots in ALPFOR and sampling sites near Furka Pass, Swiss Central Alps sampled for δ^13^C and δ^15^N natural abundances.

| Permanent plots | Latitude  (N) | Longitude  (E) | Elevation  (m) | Soil  PH | Vegetation type sampled |
| --- | --- | --- | --- | --- | --- |
| plot: 1.1 (Site-2) | 46º34’39.2” | 8º25’13.3” | 2450 | 3.2 | *Nardus stricta*-heath |
| plot: 1.2 (Site-1) | 46º34’41.8” | 8º25’15.6” | 2458 | 3.3 | *Nardus stricta*-heath |
| plot: 4.1 (Site-3) | 46º34’33.7” | 8º25’20.4” | 2409 | 4.1 | *Festuca violacea*-meadow |
| plot: 4.2 | 46º34’32.4” | 8º25’20.3” | 2386 | 3.5 | *Festuca violacea*-meadow |
| plot: 8.1 | 46º34’40.8” | 8º25’18.3” | 2440 | 3.5 | Snow-bed |
| plot: 8.2 | 46º34’37.8” | 8º25’13.1” | 2446 | 3.5 | Snow-bed |
| Site-4 | 46º34’30.2” | 8º25’09.0” | 2438 |  | Alpine grassland |
| Site-5 | 46º34’18.2” | 8º24’58.5” | 2427 |  | Alpine grassland |
| Site-6 | 46º34’12.9” | 8º24’55.8” | 2457 |  | Alpine grassland |
| Site-7 | 46º34’01.9” | 8º24’48.0” | 2482 |  | Alpine grassland |
| Site-8 | 46º33’57.5” | 8º24’50.1” | 2493 |  | Alpine grassland |
| Site-9 | 46°33'55.2" | 8°24'51.1" | 2498 |  | Alpine grassland |
| Site-10 | 46º33’45.2” | 8º24’51.3” | 2498 |  | Alpine grassland |
| Site-11 | 46º33’42.3” | 8º24’51.0” | 2458 |  | Alpine grassland |

**Supplementary Table 2.** Plant species collected at each plot in this study.

| Species | Family | Permanent plots | Abbreviation |  |
| --- | --- | --- | --- | --- |
| *Carex curvula* | Cyperaceae | P1.1; P1.2; P8.1; P8.2 | Cc |  |
| *Carex foetida* | Cyperaceae | P8.1; P8.2 | Cf |  |
| *Carex sempervirens* | Cyperaceae | P1.1; P1.2; P4.1; P4.2 | Cs |  |
| *Juncus trifidus* | Juncaceae | P1.1; P1.2; P4.1; P4.2 | Jt |  |
| *Luzula lutea* | Juncaceae | P1.1; P1.2; P4.1; P4.2; P8.2 | Li |  |
| *Luzula sudetica* | Juncaceae | P4.2; P8.1 | Ls |  |
| *Trifolium alpinum* | Fabaceae | P1.1; P1.2; P4.1; P4.2; P8.2 | Ta |  |
| *Trifolium thalii* | Fabaceae | P4.1; P4.2 | Tt |  |
| *Alchemilla pentaphylla* | Rosaceae | P8.1; P8.2 | Ap |  |
| *Campanula barbata* | Campanulaceae | P1.1; P1.2; P4.1; P4.2 | Cb |  |
| *Cirsium spinosissimum* | Asteraceae | P8.1; P8.2 | Cs |  |
| *Dracocephalum ruyschiana* | Labiatae | P4.1; P4.2 | Dr |  |
| *Gentiana acaulis* | Gentianaceae | P1.1; P1.2; P4.2; P8.1; P8.2 | Ga |  |
| *Gentiana punctata* | Gentianaceae | P1.1; P1.2; P4.1; P8.1; P8.2 | Gp |  |
| *Geum montanum* | Rosaceae | P1.1; P1.2; P4.1; P4.2; P8.1; P8.2 | Gm |  |
| *Gnaphalium supinum* | Asteraceae | P8.1; P8.2 | Gs |  |
| *Homogyne alpina* | Asteraceae | P1.1; P1.2; P8.1; P8.2 | Ha |  |
| *Leontodon helveticus* | Asteraceae | P1.1; P1.2; P8.1; P8.2 | Lh |  |
| *Leucanthemum alpina* | Asteraceae | P1.1; P1.2; P4.1 | La |  |
| *Ligusticum mutellina* | Apiaceae | P1.1; P1.2; P8.1; P8.2 | Lm |  |
| *Nardus stricta* | Poaceae | P1.1; P1.2; P8.1; P8.2 | Ns |  |
| *Nigritella nigra* | Orchidaceae | P4.2 | Nn |  |
| *Poa alpina* | Poaceae | P1.1; P1.2; P4.1; P4.2; P8.1; P8.2 | Poa |  |
| *Polygonum viviparum* | Polygonaceae | P8.1 | Pv |  |
| *Potentilla aurea* | Rosaceae | P1.1; P1.2; P4.1; P4.2; P8.1; P8.2 | Pa |  |
| *Potentilla grandiflora* | Rosaceae | P4.2 | Pg |  |
| *Ranunculus kuepferi* | Ranunculaceae | P1.1; P1.2; P8.2 | Rk |  |
| *Ranunculus montanus* | Ranunculaceae | P1.1; P1.2 | Rm |  |
| *Salix retusa* | Salicaceae | P8.1; P8.2 | Sr |  |
| *Sibbaldia procumbens* | Rosaceae | P8.1; P8.2 | Si |  |
| *Soldanella pusilla* | Primulaceae | P8.1; P8.2 | Sp |  |
| *Solidago virgaurea* | Asteraceae | P1.2; P4.1; P4.2 | Sv |  |
|  |  |  |  |  |

**Supplementary Table 3.** Foliar δ^15^N values (‰, Mean±s.e) of plant species growing with and without *Trifolium alpinum* at P1.1 and P1.2.

| Permanent plots | With  (all species) | Without  (all species) | With  (all species  without Cyperaceae and Juncaceae) | Without  (all species without  Cyperaceae and Juncaceae) |
| --- | --- | --- | --- | --- |
| P1.1 | -3.25±0.66 | -3.96±0.54 | -4.52±0.29 | -4.77±0.55 |
|  |  |  |  |  |
| P1.2 | -4.30±0.92 | -3.18±1.17 | -5.68±0.49 | -4.77±0.83 |
|  |  |  |  |  |

Non-N_2_-fixing species collected growing with and without *Trifolium alpinum* were *Carex curvula* (Cyperaceae)*, Carex sempervirens* (Cyperaceae)*, Geum montanum* (Rosaceae)*, Leontodon helveticus* (Asteraceae)*, Ligusticum mutellina* (Apiaceae)*, Luzula lutea* (Juncaceae)*, Nardus stricta* (Poaceae)*, Potentilla aurea* (Rosaceae) at P1.1 and *C. curvula*, *G. montanum, L. helveticus*, *L. mutellina*, *L. lutea, N. stricta* at P1.2, respectively.
